# Infection Rate of Respiratory Viruses in the Pandemic SARS-CoV-2 Period Considering Symptomatic Patients: Two Years of Ongoing Observations

**DOI:** 10.3390/biom12070987

**Published:** 2022-07-15

**Authors:** Gaetana Costanza, Pierpaolo Paba, Marco Ciotti, Domenico Ombres, Stefano Di Carlo, Fabbio Marcuccilli, Ada Bertoli, Loide Di Traglia, Marcello Mozzani, Lucia Piredda, Vita Petrone, Marialaura Fanelli, Carla Paganelli, Barbara Cortese, Emanuela Balestrieri, Sergio Bernardini, Massimo Andreoni, Claudia Matteucci, Antonella Minutolo, Sandro Grelli

**Affiliations:** 1 Virology Unit, Polyclinic Tor Vergata, 00133 Rome, Italy; costanza@med.uniroma2.it (G.C.); pierpaolo.paba@ptvonline.it (P.P.); marco.ciotti@ptvonline.it (M.C.); domenico.ombres@ptvonline.it (D.O.); fabbio.marcuccilli@ptvonline.it (F.M.); bertoli@uniroma2.it (A.B.); loide.ditraglia@ptvonline.it (L.D.T.); marcello.mozzani@gmail.com (M.M.); 2 Department of Laboratory Medicine, Polyclinic Tor Vergata Foundation, 00133 Rome, Italy; stefano.dicarlo@ptvonline.it (S.D.C.); bernardini@med.uniroma2.it (S.B.); 3 Department of Biology, University of Rome Tor Vergata, 00133 Rome, Italy; piredda@uniroma2.it; 4 Department of Experimental Medicine, University of Rome Tor Vergata, 00133 Rome, Italy; vita.petrone01@gmail.com (V.P.); fanellimarialaura@gmail.com (M.F.); balestrieri@med.uniroma2.it (E.B.); matteucci@med.uniroma2.it (C.M.); 5 Department of Emergency, Polyclinic Tor Vergata, 00133 Rome, Italy; carla.paganelli@ptvonline.it; 6 National Research Council-Nanotechnology Institute, 00185 Rome, Italy; barbara.cortese@nanotec.cnr.it; 7 Department of Systems Medicine, University of Rome Tor Vergata, 00133 Rome, Italy; andreoni@uniroma2.it; 8 Infectious Diseases Clinic, Policlinic of Tor Vergata, 00133 Rome, Italy

**Keywords:** respiratory viral pathogens, SARS-CoV-2 virus, surveillance research, COVID-19

## Abstract

Background: In the last two years, the SARS-CoV-2 pandemic has determined radical changes in human behaviors and lifestyles, with a drastic reduction in socialization due to physical distancing and self-isolation. These changes have also been reflected in the epidemiological patterns of common respiratory viruses. For this reason, early discrimination of respiratory viruses is important as new variants emerge. Methods: Nasopharyngeal swabs of 2554 patients, with clinically suspected Acute Respiratory Infections (ARIs) from October 2019 to November 2021, were collected to detect 1 or more of the 23 common respiratory pathogens, especially viruses, via BioFilmArray RP2.1*plus*, including SARS-CoV-2. Demographical characteristics and epidemiological analyses were performed as well as a laboratory features profile of positive patients. Results: An observational study on 2300 patients (254 patients were excluded because of missing data) including 1560 men and 760 women, median age of 64.5 years, was carried out. Considering the respiratory virus research request, most of the patients were admitted to the Emergency Medicine Department (41.2%, of patients), whereas 29.5% were admitted to the Infectious Diseases Department. The most frequently detected pathogens included SARS-CoV-2 (31.06%, 707/2300, from March 2020 to November 2021), InfA-B (1.86%, 43/2300), HCoV (2.17% 50/2300), and HSRV (1.65%, 38/2300). Interestingly, coinfection rates decreased dramatically in the SARS-CoV-2 pandemic period. The significative decrease in positive rate of SARS-CoV-2 was associated with the massive vaccination. Conclusion: This study represents a dynamic picture of the epidemiological curve of common respiratory viruses during the two years of pandemic, with a disregarded trend for additional viruses. Our results showed that SARS-CoV-2 had a preferential tropism for the respiratory tract without co-existing with other viruses. The possible causes were attributable either to the use of masks, social isolation, or to specific respiratory receptors mostly available for this virus, external and internal lifestyle factors, vaccination campaigns, and emergence of new SARS-CoV-2 variants.

## 1. Introduction

The dissemination of SARS-CoV-2 in the last two years has profoundly influenced the incidence of respiratory affections, altering epidemiological curves of other pathogens, both in pediatric and adult populations [1,2,3]. Therefore, the possibility to recognize which microorganism (Influenza A and B viruses, Respiratory Syncytial Virus, Parainfluenza viruses) is the cause of respiratory diseases, namely Acute Respiratory Infections (ARIs), is essential for monitoring and curing patients, defining therapeutic decisions, and finding adequate public health strategies [4]. The recognition is complicated by the fact that symptoms of Coronavirus Disease 19 (COVID-19) such as cough, fever, headache, with suspect of pneumonia, bronchitis, and severe respiratory failure, are similar to those of ARIs [5,6,7].

Moreover, the use of masks, social distancing, smart working, and closure of schools, as measures implemented by the national policy to contain the spread of SARS-CoV-2 (https://www.governo.it/it/coronavirus-misure-del-governo (accessed on 20 April 2022)), has further influenced the changes in the positivity rates of most respiratory viruses.

Influenza A H1N1 and H3N2 subtype viruses are two of the three combinations known to have circulated widely in humans and to currently cause seasonal influenza. These strains originated from birds and swine [5,8].

In the early 1980s, the classical swine H1N1 strain was displaced by a new European enzootic swine Influenza A viral strain: the Eurasian, avian-like H1N1 (H1avN1) lineage 1C [8]. After its rapid transmission from birds to mammals, the H1avN1 virus underwent rapid and sustained adaptation in mammals.

Before the SARS-CoV-2 pandemic, from September 2019 to February 2020, the Euro-surveillance Organization counted about 34 million flu illnesses and 20,000 deaths (www.eurosurveillance.org (accessed on 20 April 2022)). The primary immunogens of influenza virus are the two surface glycoproteins, hemagglutinin (HA) and neuraminidase (NA), which also play key roles, respectively, in the binding to and release from receptors on airway epithelial cells, which are sialic acid (Neu5Ac) containing glycans (sialoglycans) on cell surface glycoproteins and glycolipids [9].

Human parainfluenza viruses (HPIVs) are another important cause of respiratory illness in children and adults with a wide range of clinical manifestations including colds, croup, bronchiolitis, and pneumonia [10]. Seasonal HPIV virus epidemics resulted in a significant burden of disease in children and accounted for 40% of pediatric hospitalizations for lower respiratory tract illnesses (LRTIs) and 75% of croup cases [11,12]. The HPIV surface glycoprotein haemagglutinin-neuraminidase (HN) is involved in the early stages of the virus replication cycle by mediating the binding to neuraminic-acid-containing receptors through its hemagglutinin function and triggering the fusion of the virus envelope with the host cell membrane [13]. Though sensitive molecular diagnostics are now available to rapidly diagnose parainfluenza infection, effective therapies are still needed, and treatment remains supportive [10,14,15].

Human metapneumovirus (HMPV) was first isolated in 2001 from young children with symptoms of acute respiratory infections [16]. Interestingly, a well-established host immune response is evoked when HMPV infection occurs by binding the NLRP3 inflammasome that once activated will promote caspase-1-induced IL-1β and IL-18 maturation [17]. Further, infection with HMPV induces a weak memory response, and re-infections during life are common [18]. Similarly to HMPV, Respiratory Syncytial Virus (RSV) typically infects persons by 2 years of age and can cause subsequent infections throughout life [19]. The process by which RSV virions enter host cells is primarily initiated by the binding of virions to the surface molecules of host cells followed by the fusion of the virions with the host cell membrane. Annexin II, a peripheral membrane protein expressed on endothelial cells in a variety of tissues and organs, also plays a role in invasion and fusion processes [20].

With regard to coronaviruses, in 1960 the first endemic human CoV (HCoV) infection was described with the HCoV-OC43 and 229E strains [21]. Subsequently, HCoV-NL63 and HKU1 caused endemic infections in 2004 and 2005 [22]. In humans, two epidemic CoVs have been discovered in the last two decades: the Severe Acute Respiratory Syndrome (SARS-CoV) in 2003 and the Middle East Respiratory Syndrome (MERS-CoV-2) in 2012, respectively [23]. SARS-CoV was responsible for the outbreak of viral pneumonia in 2002/2003 [24].

Towards the end of 2019, in Wuhan, China, a small cluster of 41 pneumonia cases was reported, but the origin was unknown although all patients were epidemiologically linked to a seafood market of Wuhan [24].

Subsequently, epidemiological and genetic analyses led to the discovery of a novel strain of Coronavirus isolated on 7 January 2020, similar to SARS-CoV and MERS HCoV [25]. The so called SARS-CoV-2 was associated with fever, cough, and other respiratory alterations, comparable to all respiratory viruses [26], and with high mortality of old patients with comorbidities such as cardiovascular, liver, kidney diseases, or malignant tumors [27]. The S protein is a transmembrane protein that facilitates the binding of viral envelop to angiotensin-converting enzyme 2 (ACE2) receptors expressed on host cell surfaces. After attachment to the ACE2 receptor, host cell-surface proteases such as TMPRSS2 (transmembrane serine protease 2) act on a critical cleavage site on S2.38. This results in membrane fusion and viral infection [25].

Previous studies analyzed the coinfection rate of SARS-CoV-2 and influenza virus as well as that of other respiratory viruses [28]. The present study investigated the infection rate of common respiratory viruses from the pre-pandemic period, October 2019, to October 2021 in respiratory samples collected at the “Tor Vergata” Hospital of Rome (Italy), using the BioFilmArray RP2.1*plus* molecular assay. The results were compared with the incidence in previous years, considering social changes such as mask use or home isolation as well as vaccination campaigns; factors that could have influenced the results.

## 2. Materials and Methods

### 2.1. Study Design and Patients

This observational ongoing study was performed at the Virology Unit of “Tor Vergata” Hospital, Rome, Italy from October 2019 to October 2021 on samples collected mainly at the Emergency and Infectious Diseases Units. Regarding patients, the following criteria were considered: age > 18 years old; available demographic data (sex, date of birth, health insurance card); and suspicion of ARIs based on clinical symptoms and diagnosis of respiratory virus infection by BioFilmArray RP2.1*plus*. Exclusion criteria were: age < 18 years old; data missing (name or surname, date of birth, sex indeterminate); and data missing for viruses analyzed by BioFilmArray RP2.1*plus*. For patients admitted to the Infectious Diseases Unit, laboratory analysis data were collected and analyzed for epidemiological purposes. Results were tabulated and statistically analyzed. The study was conducted according to the Helsinki Declaration.

### 2.2. FilmArray Respiratory Panel 2.1plus

FilmArray Respiratory Panel 2.1plus (RP2.1*plus:* bioMérieux Clinical Diagnostics, Salt Lake City, UT, USA) rapidly and simultaneously processed 23 pathogens from nasopharyngeal swabs (NPS), as reported in Table 1. A volume of 300 μL of NPS sample was injected into a test pouch containing all necessary reagents for nucleic acid extraction, PCR amplification, and detection of the respective targets, according to the manufacturer’s instructions (BioFire Defense LLC; Salt Lake City, UT, USA) [29].

### 2.3. SARS-CoV-2 Real-Time Qualitative PCR

Up to January 2021, SARS-CoV-2 was detected using an automated liquid handling workstation for RNA extraction and PCR setup (NIMBUS, Seegene, Seoul, Korea), while amplification was carried out on the CFX96TMDx platform (Bio-Rad Laboratories, Inc., Hercules, CA, USA) using the Allplex^TM^ 2019n-CoV assay (Seegene, Seoul, Korea). The assay amplifies three viral genes: the common envelope (E) gene, the specific nucleocapsid (N), and RNA-dependent-RNA-polymerase (RdRp) gene. Results were interpreted and validated with Seegene Viewer Software [30].

From February 2021, the SARS-CoV-2 was analyzed by BIOFIRE FilmArray^®^ Respiratory Panel 2.1*plus* along with the other respiratory pathogens. Samples were collected using nasopharyngeal swabs.

#### Government Measures against SARS-CoV-2 Spread

All data and measures taken by the Italian government to contain the spread of SARS-CoV-2 are available on the government website (https://www.governo.it/it/coronavirus-misure-del-governo (accessed on 20 April 2022)). The vaccination campaign in Italy included the use of Pfizer and Moderna vaccines (98% vs. 2%) first in frail individuals, the over-70s, and health care personnel from December 2020. Over 2021, the Pfizer vaccine was administered up to age 50. Astrazeneca in school personnel and young people. But subsequently the vast majority did Pfizer, the third dose was for everyone done with this vaccine.

### 2.4. Statistical Analyses

Data were collected and organized for epidemiological and statistical analyses. Categorical variables were expressed as numbers and percentages. Positive rates were calculated as number of positive cases/total number of performed tests. For temporal comparison of positivity rates of viruses, we used an ANOVA, with *post hoc* Bonferroni test [31]. The parametric *t*-test was used to highlight differences before and after the vaccination campaign; results were expressed as mean ± SE. For association studies a *chi square test* was performed. Statistical analyses were carried out with SPSS v. 20 software and a *p* value < 0.05 was considered statistically significant.

## 3. Results

### 3.1. Clinical and Epidemiological Features of Enrolled Patients after FilmArray Respiratory Panel 2.1plus Assay

**Figure 1:**
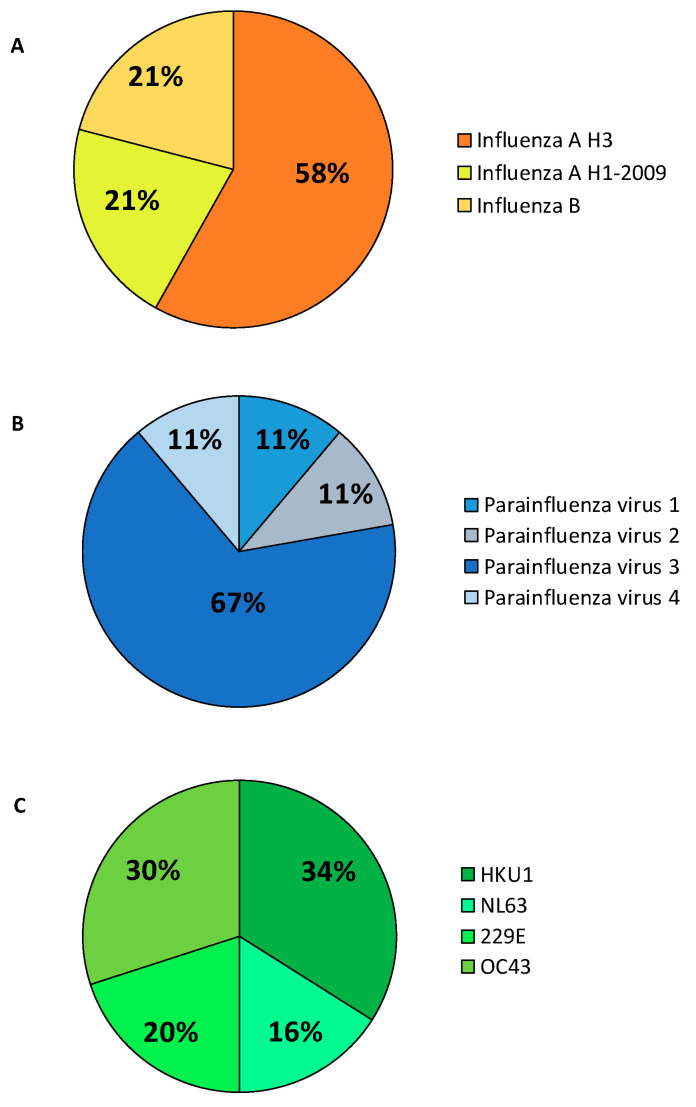
Rate of respiratory viruses detected in 2019–2021. (**A**) Influenza viruses; (**B**) Parainfluenza viruses; and (**C**) Coronaviruses.

A total of 2300 patients (period: October 2019–November 2021) with suspicious symptoms of respiratory infections were included in this study (254 patients were excluded because of data missing). Among them, 1540 were men and 760 women, with a mean of 65.4 ± 16.7 years. As reported in Table 2, 37.11% of the population analyzed resulted positive to the test. From March 2020 to November 2021, the most frequently detected pathogens included SARS-CoV-2 (31.06%, 707/2300), Inf A-B (1.86%, 43/2300), HCoV (2.17% 50/2300), and HSRV (1.65%, 38/2300). Considering the respiratory viruses research request from different hospital departments, most of the patients were admitted to the Emergency Medicine Department (41.2% of the patients), followed by the Infectious Diseases Department (29.5% of the patients). Figure 1 shows the percentages of positivity to the different Influenza viruses (Figure 1A), Parainfluenza viruses (Figure 1B), and Coronaviruses (Figure 1C).

### 3.2. Profiles of Respiratory Viruses and SARS-CoV-2 Detected in the Enrolled Patients

**Figure 2:**
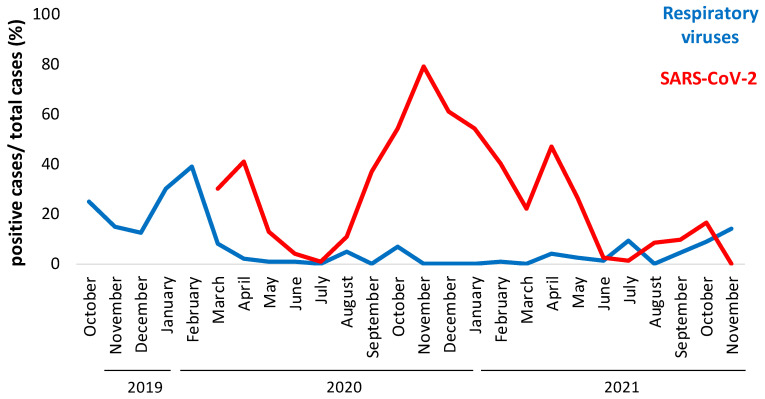
Epidemiological curve of positive cases registered every month from October 2019 to November 2021 of SARS-CoV-2 (red line) and other respiratory viruses (blue line). Samples were analyzed using BioFilmArray RP2.1*plus*. Percentages of positive cases (ordinate axis) were calculated as total number of cases analyzed every month.

We temporally analyzed the rate of positive cases for common respiratory viruses from October 2019 to November 2021 and from March 2020 (March 6, lockdown start date in Italy) to March 2021 for SARS-CoV-2 cases (number of positive cases/numbers of affected swabs). As shown in Figure 2, there was an inverse trend on the rate of positive cases considering common respiratory viruses. In fact, we observed a seasonal peak between October 2019 and March 2020 that disappeared in the period October 2020–March 2021 (Figure 2, blue line). A new inverse trend was reported in the last 4 months of 2021: the prevalence of common respiratory viruses increased, probably after the stopping of restriction measures. On the other hand, the epidemiological curve shows an increase in the prevalence of positive cases of SARS-CoV-2, especially from October 2020 to March 2021 (80% of positive detection in October 20; Figure 2 red line) and a drastic reduction in the last months, probably due to the massive vaccination measures with an attenuation of significance of respiratory pathologies.

Seven patterns of co-infection (simultaneous infection with two or more viruses) were evaluated in this surveillance research. The detailed information on these co-infection patterns is summarized in Table 3. Of the 855 positive cases found with the BiofilmArray assay, 9 (1.05%) cases were co-infected with other respiratory pathogens. The most prevalent co-infectious agents were InfA and HSRV, accounting for 8 of the 9 co-infection cases. Moreover, it was found that only 0.56% (4/707) of SARS-CoV-2 positive samples presented coinfections with other viruses, Table 3, suggesting its predominant prevalence as a mono-infection.

### 3.3. The Comparison between Percentage of Positive Cases in the Last 4 Years (2018–2021) Confirms the Influence of SARS-CoV-2

**Figure 3:**
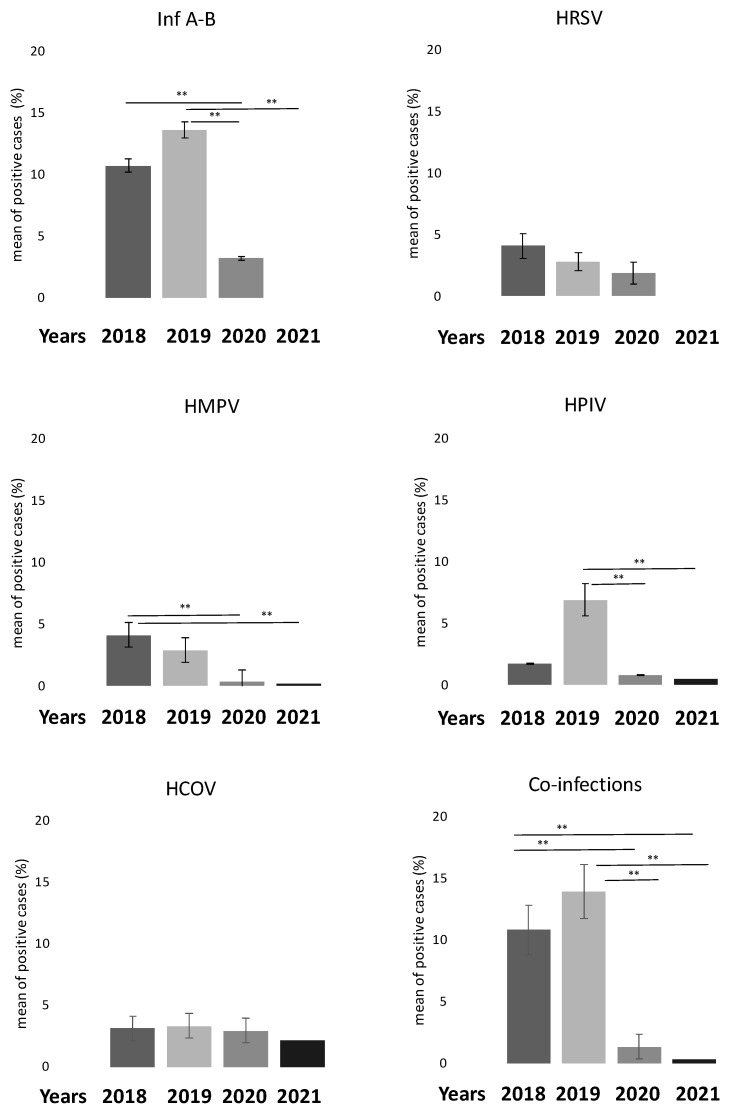
Bar graphs showing reduction in respiratory virus positivity from 2018 to 2021. Co-infection rate also decreased. ** *p* < 0.001 for ANOVA Bonferroni test. Abbreviations: HCoV: Human Coronavirus; HMPV: Human Metapneumovirus; Inf A-B: Influenza virus A-B; PIV: Parainfluenza virus; RSV: Respiratory Syncytial Virus.

We also compared the percentage of positive cases of common respiratory viruses in the last four years (2018–2021). As reported in Figure 3, the number of positive cases decreased significantly for the Inf A-B virus group (*p* < 0.001), HPIV and HMPV groups (*p* < 0.010) after 2019. In addition, we also noticed a significant decrease in the co-infection rate in the same period (*p* < 0.001).

### 3.4. SARS-CoV-2 Positivity before and after Vaccination Campaigns

**Figure 4:**
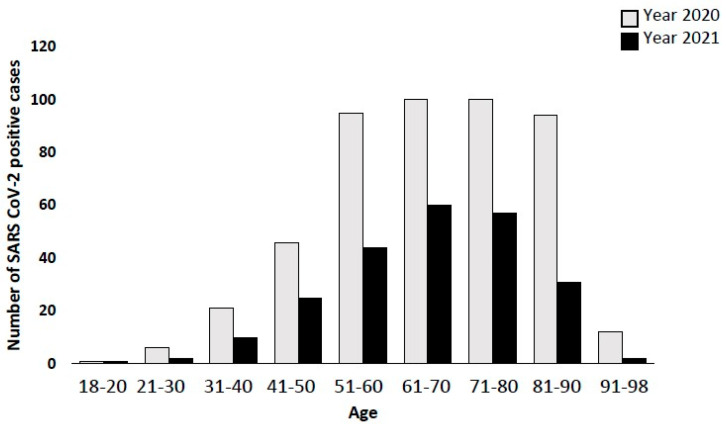
Bar graph showing a strong reduction in positive cases in 2021 compared to 2020. In particular, the Italian vaccination campaign started at the end of 2020 leading to a reduction of 64.6% of positive cases in the elderly population (>80 years old).

In order to investigate how SARS-CoV-2 positivity rates changed with implementation of Italian strategies to counteract the pandemic, such as the vaccination campaign, we analyzed the difference between positivity rates of the stratified population before and after December 2020, as reported in Table 4 and Figure 4. First, we observed a decrease in positive cases registered between 2020 and 2021 (475 vs. 232 patients). Mean age of positive SARS-CoV-2 in 2020 was 65.3 ± 3.7, whereas in 2021 it was 62.7 ± 2.8 years old. For old people (>60 aged), reduction in positive cases and rates reflected the measures adopted by the Italian government since the end of November 2020, with vaccination for the elderly (>80 years old) and fragile subjects, and consequently a decrease of 64.4% of cases. This fragile population received mainly the Cominarty (BioNTech Manufacturing GmbH, Mainz, Germany) vaccine.

### 3.5. Laboratory Features of COVID-19 Patients

Among the hospitalized COVID-19 patients, 29.7% (683/2300 patients) were admitted to the Infectious Diseases Unit. Of these, we analyzed the laboratory parameters of 444 out of 683 SARS-CoV-2-positive patients to study the effect of SARS-CoV-2 infection on such parameters. In total, 10% of the patients had other infections (Chronic Hepatitis, HIV, or bacterial infections), but none showed positivity for the other respiratory viruses using the BiofilmArray assay. As reported in Table 5, several parameters were out of the normal range regardless of the analysis group. In particular, high levels of fibrinogen, LDH, and CRP were associated with COVID-19 disease severity.

## 4. Discussion

This study represents a picture of the changes that occurred after the outbreak of COVID-19 pandemic both in epidemiological terms and in political strategies implemented by the various governments [3,32,33,34]. Our analysis on the positive rates of common respiratory viruses, in a cohort of symptomatic patients, may inform public health administrators and medical experts to aid in curbing the pandemic situation [35,36,37,38]. The emergency of COVID-19 has affected people across the globe, forcing everyone to rapidly adapt and change personal and professional life styles [39]. Starting from the first case of COVID-19 identified in Wuhan, China, in late December 2019, the outbreak has gradually spread across many countries worldwide. In the meantime, the concurrence of the peak season for respiratory tract infections, featuring severe clinical symptoms and cross-species transmission patterns, has posed a huge threat to human health [40]. Therefore, it is crucial for clinicians to timely and accurately identify patients who are very likely to have COVID-19 [41,42]. However, it is difficult to distinguish between the patients with respiratory pathologies and COVID-19, mainly since their clinical manifestations are nearly identical at the outset.

Although the etiology of ARI is complicated, some common respiratory viruses have been reported as the leading causes of the disease [1,2,28]. One constant feature of the northern hemisphere’s winter months is the circulation of influenza viruses, leading to seasonal epidemics. Along with influenza viruses, also PIVs, hMPVs, RSVs, and HCoVs were the most common causes of ARIs before the global spread of SARS-CoV-2 [10,16,43,44].

Among the common respiratory viruses with a known seasonal peak, we detected mainly the influenza viruses, parainfluenza viruses, metapneumoviruses, respiratory syncytial virus, and coronaviruses using the BiofilmArray assay. We monitored the positivity rates of common respiratory viruses and, at the same time, SARS-CoV-2 in all patients who had performed a nasopharyngeal swab for suspected infection. The samples were processed with the BiofilmArray assay method, during the 3-year period of observation and this allowed us to capture in real time the scenario of the area surrounding one of the biggest hospitals in Lazio, reflecting what was reported also in other parts of the world [3,44,45]. The spread of SARS-CoV-2 has led to a drastic reduction in the presence of other respiratory viruses, as evidenced by our epidemiological curve throughout the year 2020. As highlighted also in several studies and reports [28,32,44,46], the use of masks, social distancing measures, and a higher attention to personal hygiene such as constant hand disinfection, have resulted in an apparent disappearance of the expected seasonal peaks of many respiratory viruses, including Influenza A and Parainfluenza viruses throughout 2020 and part of 2021. Our analysis confirms the impact of pandemic on the picture of the ARI in 2020–2021, underlining the prevalence of SARS-CoV-2 as a mono-infection, with coinfection rates greatly reduced.

An increase in respiratory viruses has been taking place instead from the summer of 2021, showing in patients a more serious picture of the pathology affecting the respiratory system than SARS-CoV-2, which as the current data is showing us, is continuing to spread, but giving a less serious picture from the symptomatic point of view. Similar data were obtained in other epidemiological studies (performed outside Italy) [3,25,33,47]. We also analyzed the effect of the measures taken by the Italian government, such as vaccination campaigns. We considered January 2021 as the key time point to see the first effects of vaccination, especially in older people (over 80). We stratified the population according to age and analyzed the differences in terms of absolute numbers or percentages of positivity between 2020 and 2021.

We observed both a significant reduction among individual age groups between 2020 and 2021, and a reduction in the absolute number of asymptomatic positives among the elderly >80 years in 2021. This was attributed to the effect of the vaccination campaign.

We analyzed the laboratory parameters of hospitalized positive patients at the Infectious Diseases Unit: we highlighted that some parameters were altered such LDH, Fibrinogen, and CRP compared to the reference range. Several studies showed alteration of these laboratory features among COVID-19 patients, especially for thrombosis risk [48,49].

Our research may have some limitations as all the data were obtained from one hospital in Rome, despite the large number of individuals enrolled. Data acquired from different places would be more convincing. However, our data is supported by similar results from other studies. Moreover, other common respiratory viruses, such as rhinovirus, were not covered in this study.

## 5. Conclusions

The results could be important for many aspects (Graphical Abstract): diagnostic approaches, therapeutic choices, and health policy strategies. We concluded that in the pandemic period, particularly in the seasonal peaks of ARIs in 2020–2021, SARS-CoV-2 prevailed as a mono-infection for several reasons, among these, the ability of SARS-CoV-2 to bind with greater greed to the membrane receptors of cells of the respiratory system.

Furthermore, the timely reporting of cases, updates on clinical status and genetic predisposition of patients, the real-time analysis of data, and the appropriate dissemination of information are essential for outbreak-managing decisions.

## Figures and Tables

**Table 1:** Panel of respiratory pathogens detected simultaneously using the BIOFIRE FilmArray^®^ Respiratory Panel 2.1*plus*, including SARS-CoV-2.

Viruses	Bacteria
Adenovirus Coronaviruses (229E, HKU1, OC43, NL63) Middle East Respiratory Syndrome Coronavirus (Mers-CoV) Severe Acute Respiratory Syndrome Coronavirus 2 (SARS-CoV-2) Human Metapneumovirus (HMPV) Human Rhinovirus/Enterovirus Influenza A (H1, H1 2099, H3) Influenza B Parainfluenzavirus (PIV) 1,2,3,4 Respiratory Syncytial Virus (RSV)	*Bordetella pertussis* *Bordetella parapertussis* *Chlamydophila pneumoniae* *Mycoplasma pneumoniae*

**Table 2:** Patient cohort analyzed from October 2019 to November 2021 and FilmArray Respiratory Panel 2.1*plus* assay results.

Number of Samples	2300		
Age	65.4 ± 16.7	number	%
Sex	Male	1540	66.9
	Female	760	33.10
**Type of Infection**			
SARS-CoV-2	Positive Cases	707	31.06
Adenovirus	Positive Cases	1	0.043
Influenza viruses	Positive Cases	43	1.85
Syncytial Respiratory Virus	Positive Cases	28	1.20
Metapneumoviruses	Positive Cases	7	0.30
Parainfluenza viruses	Positive Cases	13	0.56
Coronaviruses	Positive Cases	61	2.60
	Negative Cases	1447	62.34

**Table 3:** Double and triple co-infections (October 2019–November 2021).

Type	Co-Infectious Pathogens	Number of Cases
InfA + HCoV	2	1
InfA + HPIV	2	1
InfA + SARS-CoV-2	2	1
HSRV + HCoV	2	2
HSRV + SARS-CoV-2	2	2
HMPV + SARS-CoV-2	1	1
InfA + InfB + HCoV	3	1

**Table 4:** Stratified population-based age distribution between 2020 (*n* = 475) and 2021 (232), considering SARS-CoV-2 positive rate.

Age Range	Positive SARS-CoV-2 Detection Until December 2020	Positive SARS-CoV-2 Detection Until November 2021	
	% (n. pos/n. tot)	% (n. pos/n. tot)	*p* < Value (Chi2 Test)
18–20	25 (1/4)	5.5 (1/18)	0.85
21–30	18.76 (6/32)	3.9 (2/52)	**0.02**
31–40	36.84 (21/57)	15.6 (10/64)	**0.001**
41–50	46.93 (46/98)	27.17 (25/92)	**0.004**
51–60	54–6 (95/174)	29–93 (44/147)	**0.00007**
61–70	50.76 (100/197)	27.9 (60/215)	**0.00001**
71–80	40 (100/240)	25.79 (57/221)	**0.0003**
81–90	44.84 (94/194)	19.01 (31/163)	**0.00001**
91–98	31.03 (12/29)	11.07 (2/17)	**0.03**

**Bold** for significative difference; percentage of the number of positive on total number of samples analised (% n. pos/n. tot).

**Table 5:** Laboratory features of the SARS-CoV-2-positive patients of the Infective Disease Unit.

Biochemical Data		SARS-CoV-2-Positive Cases (*n* = 444)
	Reference Values	(Mean Values ± SE)
Hematocrit	38–54	38.05 ± 0.8
Hemoglobin (g/L)	12.5–18	12.78 ± 1.2
Erythrocyte (×10^12^/L)	4–5,700,000	4.39 ± 0.27
Platelets (×10^9^/L)	150–450	226.61 ± 21.1
Leucocytes (×10^9^/L)	4–100,000	6.89 ± 2.5
Lymphocytes (%)	12–50.0	17.59 ± 4.67
Basophils (%)	0–2	0.3 ± 0.15
Neutrophils (%)	37–75	**75.85 ± 3.6**
Eosinophils (%)	0–7	0.62 ± 0.16
Monocytes (%)	3–12.0	6.67 ± 0.78
Azotemia (mg/dL)	15–50	**51.2 ± 34.7**
HDL-CHOL (mg/dL)	45–65	**29.59 ± 5.32**
CHOL (mg/dL)	160–220	147.89 ± 17.4
APTT—Seconds	20–40	30.77 ± 5.28
ATIII—Antitrombin (%)	75–129	**119.78 ± 20.85**
Fibrinogen (mg/dL)	200–400	**568.8 ± 105.29**
Gamma GT	8–61.0	47.89 ± 18.61
GOT/AST (U/L)	0–45.0	41.44 ± 9.78
GPT/ALT (U/L)	0–50.0	31.82 ± 22.12
LDH (IU/L)	122–222	**316.3 ± 75.47**
MCH (FL)	78–98	**29.1 ± 1.03**
MCHC (g/dL)	30–36	33.45 ± 0.87
MCV	80–96.0	85.9 ± 3.81
CRP (mg/L)	5–6.0; 500–1.000 ^#^	89.74 ± 29.79
PT (%)	80–120	82.71 ± 12.36
TG (mg/dL)	50–155	119.21 ± 23.45

Abbreviations: LT—alanine aminotransferase; AST—aspartate aminotransferase; APTT—Activated Partial Thromboplastin Time; Gamma GT—gamma glutamyl transferase; MCH—mean cell hemoglobin; MCHC—mean corpuscular hemoglobin concentration; MCV—mean cell volume; TP—total protein; TG—triglyceride; CHOL—cholesterol; HDL-C—high-density-lipoprotein cholesterol; LDH—lactic dehydrogenase; CRP—C reactive protein. ^#^ Inflammation range. Bold *p* < 0.05

## Data Availability

All data generated or analyzed during this study are included in this article. Further enquiries can be directed to the corresponding author.

## Abbreviations

**AdV**: Adenovirus; **CoV**: Coronavirus; **COVID-19**: Coronavirus disease 19; **HCoV**: Human Coronavirus; **HMPV**: Human Metapneumovirus; **Inf A-B**: Influenza virus A-B; **MERS**: Middle East Respiratory syndrome; **NPS**: Nasopharyngeal swab; **PCR**: Polymerase Chain Reaction; **PIV**: Parainfluenza virus; **RSV**: Respiratory syncytial virus; **SARS-CoV-2**: Severe Acute Respiratory Syndrome Coronavirus 2.
